# Consensus statement on exploring the Nexus between nutrition, brain health and dementia prevention

**DOI:** 10.1186/s12986-025-00981-6

**Published:** 2025-07-25

**Authors:** Alexandra M. Johnstone, Emiliano Albanese, Daniel R. Crabtree, Boushra Dalile, Stefanie Grabrucker, Jenna M. Gregory, Giuseppe Grosso, Adrian Holliday, Catherine Hughes, Catherine Itsiopoulos, John Mamo, Claire McEvoy, Phyo Kyaw Myint, Leticia Radin Pereira, David Vauzour, Mario Siervo

**Affiliations:** 1 University of Aberdeen, Aberdeen, UK; 2 Università della Svizzera Italiana, Lugano, Switzerland; 3 Health Determinants Research Collaboration, Aberdeen City Council, Aberdeen, Aberdeen City Council, UK; 4 Laboratory of Biological Psychology, KU Leuven, Leuven, Belgium; 5 Department of Anatomy and Neuroscience, University College Cork, Cork, Ireland; 6 Department of Biomedical and Biotechnological Sciences, University of Catania, Catania, Italy; 7 School of Biomedical, Nutritional, and Sport Science, Faculty of Medical Sciences, Newcastle University, Newcastle Upon Tyne, UK; 8 Ulster University, Coleraine, UK; 9 RMIT University, Victoria, Australia; 10 School of Population Health, Curtin University, Perth, Australia; 11 Perron Institute for Neurological and Translational Sciences, Nedlands, Western Australia; 12 Centre for Public Health, Institute for Global Food Security, Queen’s University Belfast, Belfast, Northern Ireland UK; 13 Department of Community Health Sciences, Cumming School of Medicine, University of Calgary, Calgary, Canada; 14 University of East Anglia, Norwich, UK; 15 Dementia Centre of Excellence, enAble Institute, Curtin University, Perth, Australia

**Keywords:** Ageing, Diet, Nutrition, Co-production, Mechanisms, Brain health, Dementia prevention

## Abstract

An international expert panel convened to evaluate nutrition-based approaches to brain health and dementia prevention. This consensus statement integrates perspectives from lived experiences, mechanistic evidence, epidemiology, and clinical interventions. Nutrition plays a crucial role in brain health throughout life and in cognitive decline pathogenesis, particularly through the food-gut-brain axis. Intervention effectiveness varies across the health promotion, prevention, treatment, and maintenance spectrum due to methodological differences and individual responses to nutritional interventions.

The Mediterranean and MIND dietary patterns show promise for maintaining cognitive function across studies. Multi-domain interventions like FINGER effectively combine dietary modifications with lifestyle changes to delay dementia onset in at-risk older adults. These findings align with mechanistic evidence on the food-gut-brain axis in maintaining optimal brain health by preventing neurodegeneration. Key mechanisms include gut microbiota composition and function, blood-brain barrier integrity, endothelial and mitochondrial dysfunction, insulin resistance, oxidative stress, and inflammatory processes.

Research priorities include standardizing cognitive assessment methodologies, developing early intervention strategies, and implementing integrated precision nutrition and lifestyle approaches. Incorporating patients’ and caregivers’ lived experiences in research co-production was identified as essential to support those with lived experience. The panel concluded that future directions should combine population and individual-level preventive approaches while addressing challenges in sustaining healthy behavioral changes and understanding the complex interplay between diet, lifestyle, and genetic factors in brain health and dementia prevention. Experts emphasized the need for both standardized methodologies and personalized interventions to account for individual variability in nutritional responses and facilitate effective prevention strategies across diverse populations.

**Supplementary Information:**

The online version contains supplementary material available at 10.1186/s12986-025-00981-6.

## Introduction

The importance of diet and nutrition for optimal brain health and dementia prevention has emerged as a critical area of research with significant public health implications [1]. Dementia is a significant global health challenge due to its increasing prevalence and significant economic and health care burden [2]. However, evidence suggests that up to 45% of dementia cases may be preventable through modifiable risk factors, with diet playing a central role in this prevention strategy [2]. There are multiple pathways through which nutrition influences cerebral functions and cognitive health [3, 4] including endothelial integrity, capillary inflammation, and microvascular perfusion, which are significantly modulated by nutritional and dietary factors [5]. Specific nutrients, including dietary nitrate and essential fatty acids, as well as antioxidant phytochemicals, such as (poly)phenols, have been linked to improved cognitive performance and enhanced cerebral perfusion in human studies [4, 6]. The significance of early-life nutrition on cognitive development and outcomes also highlights the importance of a life-course approach to healthy brain ageing [7]. The Mediterranean diet is proven to be cardioprotective and may also provide neuroprotection [8]. Several components of the diet, such as, a variety of plant-based foods and fatty fish, have been suggested to exert neurotrophic actions, improve vascular perfusion, and protect against neuroinflammation [9]. These potential benefits may also be partly mediated by the gut-brain axis, where dietary components modulate microbiota composition, subsequently affecting cognitive function via inflammatory and neuro-transmission pathways [10, 11]. Ongoing multi-domain interventions integrating dietary modifications with other lifestyle factors (e.g., sleep and stress management, physical activity promotion and social interaction) and cognitive stimulation, recognize the complex interplay between nutritional, lifestyle factors, and brain health [12]. Hence, nutrition and dietary factors represent key aspects to promote brain health and prevent cognitive decline and dementia risk.

## Purpose and scope

**Fig. 1:**
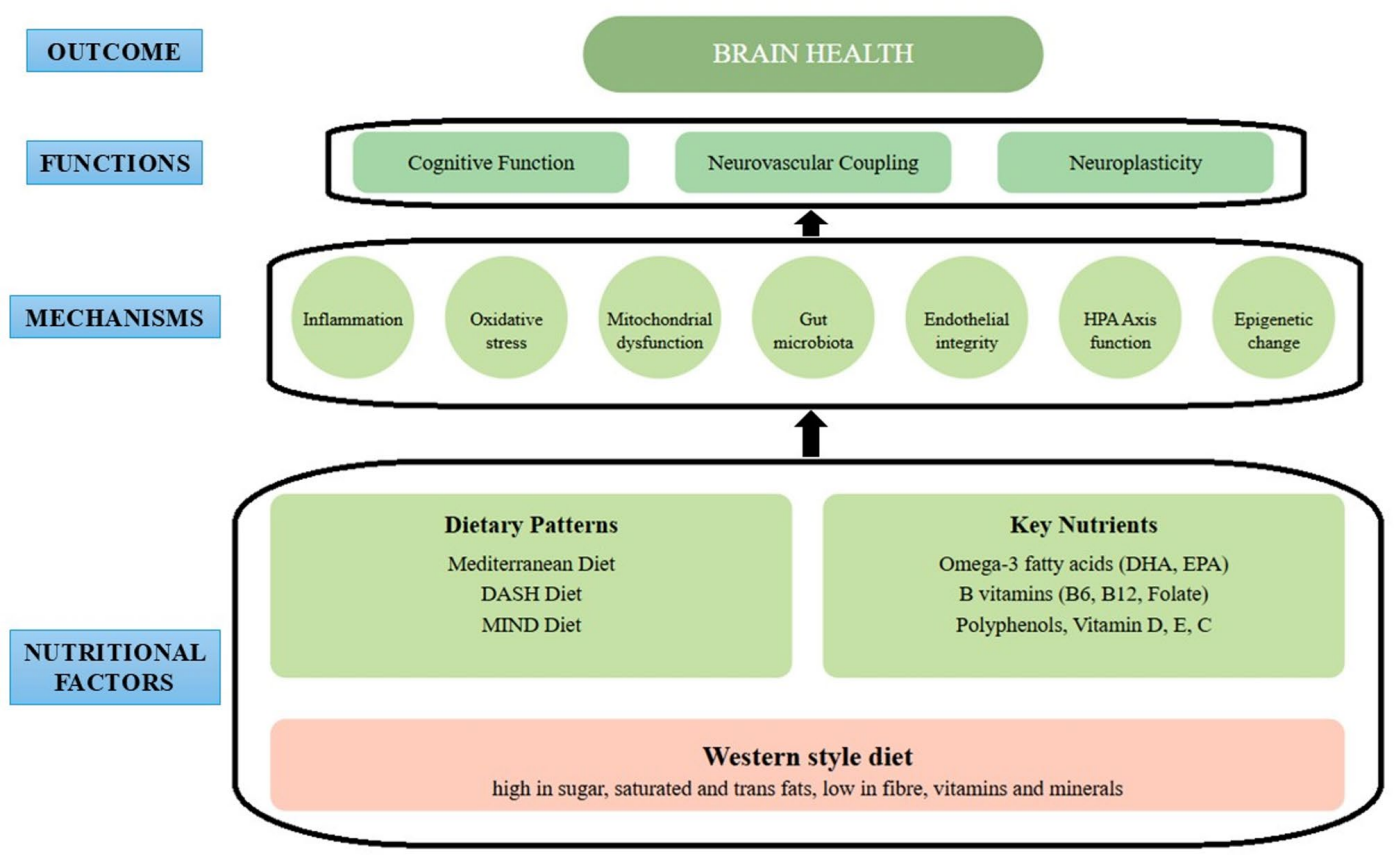
Mechanistic diagram linking nutritional and dietary factors to brain health

This consensus statement aims to provide expert opinion from multiple disciplines on the gap between scientific evidence and practical implementation of nutritional strategies for brain health and dementia prevention (see Fig. 1). The focus is on both individual and population-level approaches, recognizing that successful prevention strategies require a combination of targeted interventions for high-risk individuals and broader public health strategies. The workshop brought together leading experts in nutrition, epidemiology, geriatrics and neuroscience to address the growing global challenge of age-related cognitive decline and dementia, alongside people with lived experience of dementia. Human ethics review for clinical trials was not applicable. The workshop was held at the Rowett Institute at the University of Aberdeen on the 27-28th of June 2024. All participants gave their consent to participate in the workshop. The primary focus was to facilitate knowledge exchange and foster collaborations to better understand the relationship between nutrition and brain health, focusing on dementia prevention. The workshop was structured around six key topics, ranging from lived experience perspectives to specific dietary interventions. These included (1) exploring the burden of dementia from the perspective of carers and people living with dementia, (2) investigating biological mechanisms linking nutrition to brain health, (3) examining epidemiological evidence, (4) understanding the food-gut-brain axis, 4) evaluating and discussing evidence on nutrient-based and (5) dietary pattern-based interventions, and (6) discussing future directions and innovative opportunities for research. The format of the workshop incorporated fifteen-minute presentations followed by thirty-minute interactive sessions. A Co-Create dialog forum (https://eatforum.org/) was also used to stimulate discussion about research priorities and public health initiatives, and this interactive tool proved especially valuable in identifying challenges and proposing solutions to advance knowledge and impact in the field. The scientific discussions covered crucial areas including the role of *APO-E* genes in dementia pathogenesis and the protective effects of nutraceuticals (i.e., essential fatty acids and polyphenols). Significant attention was placed on the gut microbiota role in brain health and dietary patterns and multi-modal interventions as promising strategies for dementia prevention. A description of the scientific program of the workshop is included in Appendix 1 of the Online Supplementary Material.

## Biological mechanisms

Recent advances in understanding the biological mechanisms linking nutrition to brain health have revealed complex pathways involving vascular function, inflammation, and metabolic processes [13]. Age-related changes in cerebral blood flow, particularly alterations in capillary inflammation and microvascular perfusion, significantly influence neurodegeneration risk [14, 15]. Dietary fats have been the focus of several years of study for their potential role in affecting the risk of cognitive decline [16]. Omega-3 polyunsaturated fatty acids (PUFAs) have been extensively investigated for their broad range of effects on the human brain [17]. These fatty acids modulate immune responses to harmful stimuli and mitigate neuroinflammation by serving as precursors to pro-resolving mediators [18]. They influence nitrite oxide synthesis, reduce reactive oxygen species (ROS), and contribute to improving endothelial dysfunction, which is commonly observed in certain forms of dementia [19]. Additionally, omega-3 PUFAs play a crucial role in maintaining the structural integrity of the brain by preserving the blood-brain barrier, combating brain atrophy, promoting neurogenesis, and preventing the age-related decline in volume of brain regions involved in cognitive functions [20, 21], such as the hippocampus [22].

Alzheimer’s disease (AD) has been identified as having a substantial post-prandial component, with also other dietary fats playing a crucial role in amyloid plaque synthesis [23]. Numerous studies have shown that low status of the B-vitamins (folate and vitamin B12 and to a lesser extent vitamin B6) and / or elevated plasma homocysteine are associated with cognitive decline and dementia [13]. These B-vitamins are involved in one-carbon metabolism and the re-methylation of homocysteine to methionine, the precursor of the universal methyl donor S-adenosylmethionine [24]. Disruption of methylation processes could alter neurotransmitter production and disrupt gene expression within the Aβ pathway and lead to hyperphosphorylation of tau [25]. Furthermore, low B-vitamin status and /or elevated plasma homocysteine has been associated with white matter lesions, neurofibrillary tangles and increased rates of brain atrophy [26]. Most importantly, there is randomized controlled trial evidence demonstrating that B-vitamin supplementation can reduce the rate of brain atrophy in patients with Mild Cognitive Impairment [27].

The *APOE4* genotype has been confirmed as a significant risk factor for AD increasing the risk by up to 12-fold in homozygotes, primarily through its impact on lipoprotein clearance from blood [28, 29]. Investigations comparing different meal compositions, particularly saturated fats versus olive oil, in individuals with varying *APOE* alleles have provided insights into genotype-specific responses to dietary interventions [30]. These findings suggest potential pathways for personalized nutritional approaches based on genetic profiles. The role of bile acids has also emerged as particularly significant, with evidence showing their ability to cross the blood-brain barrier and influence brain function [31].

The role of insulin resistance in cognitive decline has emerged as a significant area of investigation, with particular attention to the interplay between glucose sensitivity and lipid metabolism [32]. Impaired glucose sensitivity, often associated with insulin resistance, can lead to suboptimal glucose utilization in the brain, resulting in energy deficits that negatively impact cognitive performance [33]. This impairment is particularly pronounced in the context of age-related cognitive decline, where reduced glucose uptake in regions like the hippocampus has been linked to memory deficits and decreased cognitive flexibility [34]. Furthermore, chronic dysregulation of glucose metabolism has been implicated in the development of AD, where glucose hypometabolism in the brain precedes the onset of clinical symptoms and amyloid-beta accumulation [35]. Insulin resistance often leads to alterations in both glucose and lipid metabolism, creating a metabolic environment that is unfavorable for brain health: increased levels of circulating free fatty acids, often seen in insulin-resistant states, can further impair glucose utilization in the brain, while simultaneously promoting neuroinflammation and oxidative damage [36]. Additionally, elevated lipid accumulation, particularly in the form of cholesterol and triglycerides, has been associated with the pathogenesis of AD, as dysregulated lipid metabolism can impair neuronal membrane integrity and disrupt synaptic signaling [37].

Other dietary components that are currently investigated for their potential role in brain health are (poly)phenols. Such naturally occurring compounds found in plant-based foods exhibit a wide array of biological activities that may influence brain health through their antioxidant and anti-inflammatory properties [38]. (Poly)phenols may also influence brain plasticity by promoting neurogenesis, particularly in regions critical for learning and memory [38]. Furthermore, polyphenols have been observed to modulate the signaling pathways involved in amyloid-beta (Aβ) aggregation, a key feature of Alzheimer’s pathology [39]. Observational studies support the hypothesis that higher (poly)phenol intake would be associated with lower risk of cognitive decline [40]. Human trials investigating (poly)phenols, particularly anthocyanins, have shown promising results in cognitive performance enhancement [41], with MRI studies confirming improved brain perfusion [13]. To date, it is still unclear whether these molecules can directly exert the observed benefits by passing the blood-brain barrier (although absorbed in minimal quantities) or rather provide indirect positive effects via modulation of the gut microbiota and the resulting metabolites improving vascular and endothelial health [40, 42, 43].

Among the most studied gut metabolites is trimethylamine N-oxide (TMAO), produced from seafood, egg, and whole-grain products [44], and its relationship with AD, with elevated TMAO levels correlating with increased AD risk [45]. However, considering that TMAO may depend on the consumption of healthy foods, its use as a biomarker predictor of disease is considered controversial [46].

A case study of blue-sky research revealed novel biomarkers for early Amyotrophic Lateral Sclerosis (ALS) detection and management, establishing a translational research framework where the successful identification of metabolic and inflammatory biomarkers in ALS could guide similar investigations in early-stage dementia, potentially informing the design of new studies to uncover neuronal protection pathways and nutritional intervention targets before the onset of cognitive deficits. The research highlighted TDP-43 as a crucial protein biomarker for the early detection of ALS [47]. The discovery that TDP-43 can be detected in stool and blood samples could make screening more accessible and allow for wider population screening and earlier intervention opportunities, using more sensitive and specific tools [47, 48]. Therapeutic interventions targeting gut TDP-43 have shown significant promise, with clinical studies reporting improved motor function and extended survival rates [49].

Circulating blood biomarkers for Alzheimer’s and dementia, including plasma amyloid-β, phosphorylated tau (p-tau217, p-tau181), neurofilament light chain, and glial fibrillary acidic protein (GFAP), represent a rapidly advancing field with significant potential for minimally invasive early detection of dementia [50]. Recent studies demonstrate these markers correlate well with PET imaging and cerebro-spinal fluid biomarkers while offering greater accessibility and scalability for screening at-risk populations [51, 52]. This important topic was not fully addressed during in the workshop but will be included in the next expert panel workshop.

## Epidemiological evidence

Prevention and treatment of dementia represent a critical public health priority, with evidence suggesting that dietary factors may play a preventive role in cognitive impairment [53]. Comprehensive evidence syntheses of the literature on individual food groups showed the potential preventive role of certain dietary elements, such as fruit and vegetable [54], fish [55], wine [56], and poultry [57], while an increased risk of cognitive impairment for excess intake of total and red processed meat [57]. Furthermore, dietary fiber– an essential component of any healthy dietary pattern whose recommended intake is not met by individuals living in Western societies– may be critical in preventing cognitive decline. Higher dietary fiber intake is associated cross-sectionally with better cognitive function in older adults [58, 59] and is longitudinally inversely associated with risk of disabling dementia, such that individuals with the lowest dietary fiber intake were at a 26% increase risk of developing dementia over 20 years follow-up [60]. In line with this, evidence from observational studies published over the last decade has shown an inverse relation between higher adherence to plant-based dietary patterns, such as the Mediterranean diet, and occurrence of cognitive decline [61]. Higher adherence to the Mediterranean diet has been associated with decreased risk of cognitive impairment, dementia, and AD by 10–20% (depending on the statistical model) [62]. The Mediterranean diet is characterized by a variety of specific dietary and culinary features, including richness in fruit and vegetables, whole grains, legumes, nuts, oily seeds and olive oil as major sources of fats, alternative consumption of animal sources of protein (dairy, fish, poultry, eggs), limited consumption of red meat and moderation with wine during meals [63]. Such features are to be contextualized within the cultural heritage of Mediterranean populations, alongside with a long history of culinary techniques (i.e., slow cooking, frequent use of spices and herbs for sofrito, frugal meal preparation), conviviality, and a strong embedding with the local natural environment that may all, in some way, be also responsible for a healthier aging and better cognitive health [64, 65]. An internationally applied plant-based dietary pattern is the DASH (Dietary Approach to Stop Hypertension) diet, which emphasizes sodium reduction and lacks of some typical elements of the Mediterranean one (such as olive oil), have also demonstrated an association with lower risk of cognitive impairment and dementia [66]. Lately, the MIND (Mediterranean-DASH Diet Intervention for Neurodegenerative Delay) diet has been proposed as an international pattern that incorporates features of both the original dietary models with emphasis on specific components derived from the most compelling findings in the nutrition-brain field (i.e., beneficial effects of green leafy vegetables, particular types of fruits such as berries, inclusion of nuts over legumes– only beans are considered– or detrimental effects of fried food) [67]. The majority of observational studies showed that the MIND diet is associated with better global cognitive function and possibly specific domains of cognition in older adults [68] as well as 17% lower risk of dementia (in individuals more adherent) [69].

The World Health Organization’s perspective on brain health emphasizes a life-course approach, drawing on the Barker’s hypothesis which suggests that in-utero conditions influence long-term health outcomes [70]. Neuroplasticity and brain mass development during childhood, followed by neural loss in older adults, highlight the importance of early intervention strategies [71]. The structural and functional roles of fats in brain health underscore the complexity of nutritional requirements for optimal cognitive function [72]. In consideration of policy, the Swiss Brain Health Plan 2023–2033 [73] exemplifies a comprehensive approach to brain health promotion. The plan advocates for upstream thinking and whole-population level approaches while maintaining targeted interventions for high-risk groups [73]. This dual strategy aims to reduce health inequalities through both passive engagement for nudged behavior change and active early detection programs [73]. Future directions in dementia research emphasize the need for new technologies in assessing dietary intake and cognitive function, with artificial intelligence offering promising opportunities for identifying effective interventions. Integrating individual and universal approaches, combined with sustainable food systems, represents the most promising path forward. Success will require coordinated effort at both individual and policy levels, with particular attention to creating sustainable, long-term solutions for brain health promotion.

## Nutrient-based intervention studies for dementia prevention

Current research into nutrient-based interventions for cognitive improvement and dementia prevention reveals complex challenges and opportunities. Evidence suggests that several key nutrients including omega-3 fatty acids, B-vitamins and vitamin D can protect against cognitive decline and dementia but the results are not conclusive [53]. There is substantial epidemiological evidence reporting that omega-3 fatty acids, particularly DHA and EPA are important for cognitive health and are associated with a lower risk of cognitive decline and dementia [74]. One recent meta-analysis of clinical interventions reported that omega-3 fatty acid supplementation had neuroprotective effects improving general cognition and executive function in older individuals [75]. Low status of the B vitamin (particularly folate, vitamin B12 and vitamin B6) involved in one-carbon metabolism have been consistently associated with an increased risk of cognitive decline and dementia in epidemiological studies [76]. The Trinity-Ulster-Department of Agriculture (TUDA) study has provided valuable insights into the role of B vitamins in cognitive function, demonstrating that individuals with low B6 or riboflavin status exhibit poorer cognitive performance [77]. Randomized controlled trials have reported beneficial effects from B vitamin supplementation particularly in vulnerable groups such as those with MCI or suboptimal B-vitamin status. Of note, the strongest RCT evidence is from the VITACOG trial, which demonstrated that B-vitamin supplementation significantly slowed cognitive decline and reduced the rate of brain atrophy in participants with MCI, with the greatest benefits observed in those with elevated plasma homocysteine [27, 78]. Evidence on other specific vitamins, including antioxidants such as vitamins C and E, is generally weaker [79], although a recent quantitative overview of the literature unveiled the potential significance of lower vitamin concentrations in individuals with Alzheimer’s disease [80]. Flavonoids from berries and other (poly)phenols demonstrate cognitive benefits [81] and vitamin D adequacy correlates with better cognitive function [82]. Research indicates that combined nutrient approaches may be more effective than single-nutrient interventions.

While epidemiological evidence consistently indicates that nutrition is important for cognitive health, the findings from RCTs has been less consistent [82]. An area of increasing interest is personalized nutrition, which recognizes that individual differences in genetics, microbiome composition, metabolic profiles, and lifestyle factors can affect nutrient metabolism and response. Personalized nutrition approaches tailor dietary interventions to these individual characteristics, potentially enhancing cognitive outcomes and delaying cognitive decline. Incorporating such tailored strategies into future research may improve the effectiveness of nutrient-based interventions for dementia prevention, moving beyond a ‘one-size-fits-all’ model [83–85].

Recent findings highlight important diet–gene interactions. For example, Ebright et al.. in 2024 examined DHA metabolism among *APOE4* carriers [86], demonstrating altered uptake and utilization patterns that may influence responsiveness to DHA supplementation. Similarly, Liu et al.. in 2024 [87] investigated the interaction between dietary fat intake and *APOE4* status, showing that high-fat intake may exacerbate cognitive decline in *APOE4* carriers. These findings underscore the complexity of nutritional modulation in the context of *APOE* genotype and highlight the need to tailor dietary interventions accordingly.

The field must acknowledge that absence of statistically significant results does not necessarily indicate a lack of effect. This is particularly relevant given the unique challenges faced by nutritional intervention trials, that pharmaceutical studies do not encounter. The nutritional status of the population is of paramount importance, as many interventions have been conducted in groups with optimal nutritional status, making them less likely to benefit from the intervention. Supplementation studies report the greatest benefits in individuals with existing deficiencies, rather than those with adequate nutrient status, underscoring the importance of baseline nutritional assessment [1]. Additionally, the timing and life stage of the intervention are important considerations as the strategies that prevent dementia in later life may not be as effective as they are in midlife [1]. Other important considerations include duration of intervention as cognitive changes occur slowly over time, sample size and statistical power, dose of nutrient exposure and outcome measures [1]. Finally, a various interventions are tested in healthy individuals with normal cognitive performance. However, to better understand the magnitude of the intervention’s efficacy, studies with challenge models could be implemented in healthy participants, particularly to help gauge the ability of an intervention in preventing cognitive impairment or decline, and promoting cognitive resilience [88]. Examining the effects of nutritional interventions could be done in healthy populations exposed to cognition-taxing factors including acute or chronic stress, poor sleep outcomes, or increased sedentary behavior [88]. Identifying vulnerable populations that are either subclinical or that struggle chronically or periodically with one or more cognition-taxing factors could help better uncover the boundary conditions for effectiveness and preventative capacity of various nutritional interventions in relation to cognitive decline and dementia [1]. Nutrient interactions add another layer of complexity, as they can work synergistically or antagonistically, making isolated nutrient studies particularly challenging to interpret. The *APOE* genotype has become crucial in determining individual responses to nutritional interventions. The *APOE* genotype affects DHA status and is thought to influence the response of cognitive outcomes to intervention but the results are not consistent [74]. There is small amount of evidence indicating that the *APOE E4* could be influenced by B-vitamin status, with studies reporting poorer cognitive function in *APOE E4* carriers but this warrants further investigation [89, 90]. This may further promote the development of personalized nutrition approaches for brain health.

Methodological challenges in conducting nutrient supplementation research are substantial [91]. The field lacks appropriate reference data for many populations, and establishing hard clinical endpoints proves difficult. Traditional dietary assessment methods, such as food frequency questionnaires and 24-hour recalls, are inadequate for this population, because they rely on retrospective recall methodology [92]. While supplementation studies apply prospective dietary assessment techniques to offer more controlled conditions, they must still account for background diet variations. In this context, the feasibility of randomized controlled trials has been questioned, leading to suggestions for alternative approaches using graded evidence assessment methods. The focus has shifted toward demonstrating marginal gains in cognition and delayed decline rather than absolute improvements [93]. This approach may prove more realistic and meaningful for intervention assessment.

Methodological challenges in assessing cognitive function remain also significant [94]. Current discussions focus on developing more sensitive measurement tools and standardizing assessment approaches. The variability in test-retest reliability and the need for disease-specific assessment tools have been highlighted as crucial areas for improvement [94]. Furthermore, the relatively small treatment effect sizes observed in pharmaceutical interventions (approximately 2.3%) underscore the importance of preventive approaches, mainly targeting individuals in their third and fourth decades of life [93, 95].

From a lived-experience perspective, while interventions may aim to improve cognitive function, they can also have unintended effects on quality of life, such as changes in eating habits, social interactions, or financial burden.

## Dietary patterns for brain health and multi-domain interventions

Translating epidemiological findings into nutritional interventions is a promising strategy for the prevention of cognitive decline [96]. Research indicates that dietary patterns offer more comprehensive benefits than single-component approaches. The Med-Ex trial [97] has demonstrated significant improvements in global cognition and memory after just six months of improved diet quality. An important finding is that undernutrition and weight loss often precedes dementia diagnosis by a decade or more, suggesting a critical window for nutritional intervention [98]. The PROMED-EX trial [99], focused on ‘Protein Enriched Mediterranean Diet & Exercise’ showed significant reversal of undernutrition and improved cognition in response to a personalized high protein Mediterranean diet intervention among older community dwelling adults at risk of both undernutrition and cognitive decline. A few studies conducted in the context of the PREDIMED (*PREvencion con Dieta MEDiterranea*) in Spain [100, 101] showed improvements in global cognition, memory, phonetic fluency in the intervention groups administered to follow a Mediterranean diet compared to a control one. However, a trial conducted in Australia [102] reported null findings on potential improvements of adoption of a 6-month Mediterranean-type diet compared to a control diet on global cognition, memory, processing speed, or visual-spatial memory. The MIND (Mediterranean-DASH Intervention for Neurodegenerative Delay) diet based on evidence from the diet-dementia field, has been associated with slower rates of cognitive decline [103] and reduced risk of AD [103]. A 3-year randomized trial compared the MIND diet with a control diet in 604 cognitively healthy adults with family history of dementia and both groups were on a mild caloric restriction plan. No significant differences were observed between groups in cognitive scores or brain MRI outcomes, suggesting cognitive benefits in this study may be the result of caloric restriction rather than the specific dietary pattern [104].

The FINGER (Finnish Geriatric Intervention Study to Prevent Cognitive Impairment and Disability) trial has set a new standard for multi-domain interventions in dementia prevention [105]. This groundbreaking study combined dietary modification with exercise, cognitive training, and vascular risk monitoring. Its success has led to the World-Wide FINGERS initiative, adapting the intervention model for different cultural and healthcare contexts globally [12]. This adaptation demonstrates the potential for scaling effective interventions across diverse populations while maintaining cultural sensitivity [106]. Research has identified heterogeneity in the diet quality and eating behaviors of different racial and ethnic populations [107]. Furthermore, the association between certain dietary patterns, such as high whole grain consumption, and improvement in cognitive outcomes may be race-specific [108, 109]. Therefore, it has been proposed that nutrition-related strategies that incorporate cultural sensitivities may prompt greater acceptance and bring about long-term sustainable change [110, 111].

Adapting the Mediterranean diet to different cultural contexts with a multi-domain approach has proven successful, as demonstrated in Australian studies. The Medwalk [112] and LIILAC (Lifestyle Intervention in Independent Living Aged Care) [113] trials have provided compelling evidence for combining Mediterranean diet with exercise interventions, demonstrating significant improvements in memory and attenuation of cognitive decline. Notably, participants showed substantial increases in Mediterranean diet adherence scores after 12 months, suggesting the intervention’s sustainability [113]. Other ongoing intervention trials following the line of action of the FINGER approach include the FINGER-NL [114], the Heritage Study [115], the AU-ARROW (Approach to Reduce Dementia Risk by protecting brain health with lifestyle intervention study) [116], the CAN-THUMBS UP (Canadian Therapeutic Platform Trial for Multidomain Interventions to Prevent Dementia) [117], the LatAm-FINGERS (Latin American Initiative for Lifestyle Intervention to Prevent Cognitive Decline) [118], and the SINGER (The SINgapore GERiatric Intervention Study to Reduce Cognitive Decline and Physical Frailty) [119], and the U.S. POINTER study (The *U*.*S*. study to *p*r*o*tect brain health through lifestyle *inte*rvention to *r*educe risk), which is a large-scale 2-year RCT adapting the FINGER interventions to American culture [120], are currently testing the adaptability of the multidomain lifestyle intervention globally.

The success of these multi-dimensional approaches highlights the importance of considering both nutritional and lifestyle factors in dementia prevention. While dietary intervention remains central, integrating exercise, behavioral support, and cultural adaptation appears crucial for optimal outcomes. The combined evidence from the observational studies and the multidomain intervention trials suggests that future intervention strategies should adopt such a holistic line of action, considering the nutritional aspects and the broader lifestyle and cultural context of the target population. This comprehensive approach, incorporating elements of both the Mediterranean and MIND diets within a multi-domain intervention framework, represents the most promising direction for dementia prevention research and practice. Some individuals might find it either financially or practically difficult to incorporate foods like fish, berries, and leafy greens into their diet, leading to challenges in following a Mediterranean or MIND diet. By understanding the lived experiences of individuals participating in nutrient-based interventions, researchers and clinicians can develop more effective and personalized approaches to dementia prevention.

## A hot topic: The food-gut-brain axis research for a healthy ageing brain

The relationship between gut microbiota and brain health has become a critical area of research in understanding dementia pathogenesis and advancing precision medicine for prevention. Emerging evidence highlights the significant interplay between the gut and brain, particularly in AD and behavioral and psychological symptoms of dementia (BPSD) [10]. Studies suggest that the gut microbiome can change rapidly, often within days of dietary modifications, and that early life events leave a lasting imprint on its composition. Aging is associated with distinct changes in gut microbiota composition, contributing to “inflammaging”– a state of chronic, low-grade inflammation that exacerbates age-related cognitive decline [121].

Dietary fiber plays a central role in regulating microbiota diversity, which has profound implications for cognitive health [122]. Groundbreaking research including fecal microbiota transplantation (FMT), has provided compelling evidence of the gut-brain connection [122]. In AD patients, gut dysbiosis has been identified, with an overrepresentation of inflammation-promoting bacteria linked to cognitive decline [10]. Animal studies show that transferring AD patient microbiota to rodents results in cognitive impairments, altered colon morphology, and reduced neurogenesis, suggesting that cognitive decline can occur independently of amyloid plaque deposition [123]. Gut microbes influence hippocampus-dependent learning and plasticity by regulating neurotransmitters and immune molecules [124]. Studies in germ-free mice indicate that gut bacteria impact hippocampal structure and neuronal function [125]. Recent research has also highlighted a genetic link between gastrointestinal disorders and dementia, suggesting shared risk factors [126]. Microbiota shifts are especially evident in the early stages of cognitive decline, particularly in those with mild cognitive impairment (MCI), which often precedes AD [127].

These microbial changes affect host metabolic pathways, influencing cognitive function. Metabolites like short-chain fatty acids (SCFAs) produced by gut bacteria, enter the bloodstream and impact brain health via a variety of signaling pathways including direct and indirect signaling pathways [128]. Direct signaling pathways of SCFAs include their free-fatty acid receptors 2 and 3 and inhibition of histone deacetylases, which are enzymes involved in epigenetic modifications that negatively regulate long-term memory formation synaptic plasticity. Indirect signaling pathways include the vagus nerve, (neuro)inflammation, hormone release, as well as promoting gut- and blood-brain barrier integrity [128, 129]. Additional metabolites of relevance to cognition include tryptophan derivatives [129]. Studies have shown that these microbial compounds can predict cognitive decline in AD [130].

Notably, manipulating gut microbiota in animal models can improve memory and cognitive function, with fecal transplants from healthy mice showing positive effects in AD models [131, 132].

The emerging field of psycho-biotics, distinct from prebiotics and probiotics, has gained attention as a potential therapeutic approach for cognitive health. Metabolomic analyses have identified key microbial pathways, such as those involving tryptophan, guadinobutyric homocitrulline, and taurine, which are mediators of the gut-brain axis [133].

While most of these findings have been explored in clinical experimental settings, the practical challenges in sample collection from ageing populations remain. Additionally, biomarkers like TDP-43, has shown promise for early identification of neurodegenerative conditions like ALS, with gut-targeted therapies demonstrating potential improvements in motor function and survival [48].

High-fiber dietary interventions have emerged as a promising therapeutic strategy, highlighting the potential for dietary modification in supporting cognitive health through modulation of the gut-brain axis. High-fiber dietary interventions have emerged as complementary therapeutic strategies for ALS, supporting gut health and potentially moderating disease progression [134]. This gut-brain axis approach represents a fundamental shift in ALS treatment paradigms, suggesting that early dietary modifications combined with targeted therapies could significantly improve patient outcomes [134]. This case study demonstrates how discovering accessible biomarkers and dietary interventions could guide similar approaches for early detection and management of other neurodegenerative conditions (i.e., Dementia, Parkinson’s Disease) and impact on disease prevention through gut microbiota modification and targeted nutritional therapies.

As the field of dementia research shifts toward precision medicine, there is increasing emphasis on metabo-typing and analyzing individual responses to interventions. A key area of focus is standardizing assessments of diet, lifestyle, and hydration’s impact on neurodegenerative disease progression. However, translating these personalized approaches into large-scale public health interventions remains challenging. High interindividual variability, the need for advanced analytical infrastructure, and the complexity of capturing dynamic biological and lifestyle data across diverse populations can limit feasibility. Moreover, cost, accessibility, and equity considerations must be addressed to ensure that precision strategies do not exacerbate health disparities [135].

Research into anorexia of ageing has revealed complex relationships between gut health and appetite control through hormonal mechanisms [136]. Studies show that older adults with low appetite exhibit amplified feeding hormones, potentially leading to hypersensitivity and increased malnutrition risk [137]. This results in more nutrients reaching the distal gut, which has implications for overall nutrition status. Using organoids has helped identify individuals at risk of low appetite through ad libitum protocols [138].

Investigation of prebiotics’ effects on cognitive reserve shows varying results across studies, with differences in duration, sample size, and cognitive domains creating challenges in interpretation [88, 139]. Limited studies exist in clinical or subclinical samples, with the existing findings yielding limited efficacy and mostly generalizable to cognitive-healthy individuals.

While randomized controlled trials remain the gold standard for evidence, comparisons between single and multiple prebiotic supplementations continue to pose challenges in interpretation. Nevertheless, there is increasing recognition of the need for precision-targeted interventions that account for diet, lifestyle, and gut health. Factors like stress, sleep patterns, sedentary behavior, and diet quality interact with the gut microbiome and can together or independently to shape cognitive trajectories, suggesting that personalized interventions that promote cognitive resilience will likely determine the greatest benefits [88].

## Lived experience perspectives: lifestyle, social aspects, and economic factors in nutrition management

The testimonies of individuals living with dementia and their carers provided powerful insights into the daily challenges of managing nutrition and maintaining quality of life. Their experiences highlighted the complex interplay between dementia, dietary habits, and overall well-being. Participants emphasized the importance of plant-based diets with reduced meat consumption, hoping such dietary modifications might slow disease progression. Historical perspectives on food accessibility and consumption patterns revealed significant changes from the 1950s to the present day. The shift from daily shopping at local greengrocers to supermarket-dominated food systems has impacted food quality, availability, and waste. The entry into the European Community in 1973 marked a crucial transition, introducing more packaged foods and standardized produce. Carers shared experiences of managing changing food preferences and unusual eating behaviors in dementia patients, emphasizing the need for nutrition education from childhood. The challenges of food delivery services in rural areas were highlighted, particularly their failure to consider the specific needs of aging populations. The importance of inter-generational knowledge transfer about nutrition emerged as a key theme, with grandchildren often encouraging older adults to try different foods. Physical activity was emphasized as crucial for healthy ageing, with participants noting the importance of group activities for social connection and maintaining core strength and flexibility. The pandemic’s impact on formal exercise classes highlighted the need for alternative approaches to staying active. Remote and rural communities face particular challenges regarding food accessibility, with transportation, disruptions to food delivery services and seasonal tourist influxes affecting the availability and affordability of healthy, fresh produce [140, 141]. Modern solutions such as food banks and local food larders have emerged as important resources, offering variety and reducing waste through smaller pack sizes, though stigmatization may negatively impact engagement [141, 142]. The role of media personalities and social platforms in shaping dietary habits was noted, along with the need for academics to communicate health messages to the public effectively. The testimonies of those with lived experience emphasize the importance of understanding the intricate relationship between the social determinants of health - broadly defined as the conditions in which people are born, grow, live, work and age and diet and health outcomes, to ensure the implementation of equitable population-level interventions [143].

Eating and swallowing challenges in patients with dementia may represent some of the most distressing aspects of daily care [144]. Carers may report rapid and significant changes in food preferences and onset of food cravings [145]. Physical challenges may impact on quality of life as as individuals struggle with using utensils, recognizing food, or coordinating chewing and swallowing movements [146]. These eating difficulties increase the risk of unintentional weight loss and nutritional deficiencies and create substantial caregiver burden and stress [147, 148]. Structured assessment approaches involving healthcare professionals - including speech therapists, dietitians, and occupational therapists - provide essential support for carers to identify early warning signs and implement effective nutritional strategies to maintain quality of life and nutritional status [149, 150].

## Identification of future research priorities using the CO-CREATE TOOL

**Fig. 2:**
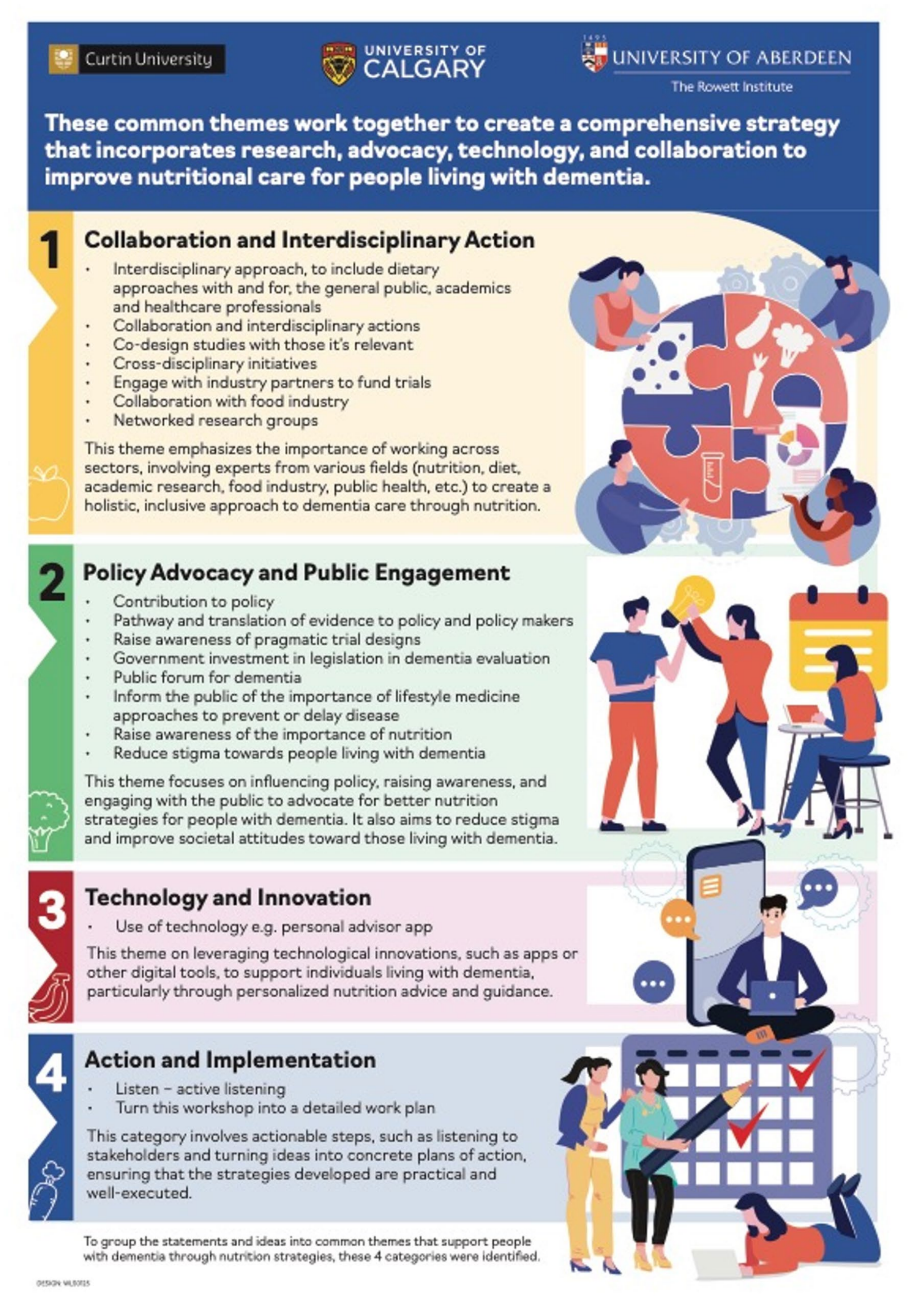
Outcomes from the co-create session

The CO-CREATE session proved instrumental in identifying and prioritizing future research directions in nutrition and brain health and the outcomes are summarized in the infographic in Fig. 2. The collaborative discussion revealed several interconnected priority areas, emphasizing the need for both fundamental research and practical implementation strategies. Early detection and prevention emerged as primary focuses, particularly on developing nutritional strategies to delay AD onset. The need for improved cognitive assessment methods was highlighted, alongside the importance of closing the gap between scientific knowledge and practical application in nutrition and brain health. Women’s health emerged as a distinct priority area, recognizing the unique impacts of female-specific factors, such as pregnancy, on brain health outcomes. The integration of lived experience in study design was identified as crucial for ensuring research relevance and effectiveness, particularly in understanding and addressing the barriers older adults face in maintaining adequate nutrition.

Methodological considerations formed another key priority cluster, emphasizing the need for robust statistical power, appropriate sample sizes, and standardized mechanistic assessments of dietary interventions. Evidence-based approaches were consistently emphasized, particularly in developing sustainable solutions that can work at a population level while ensuring equitable access to information and resources. Understanding the mechanistic effects of diet and lifestyle on disease progression was identified as crucial for developing effective interventions. Contemporary research emphasizes free-living assessment approaches, utilizing tools such as continuous glucose and blood pressure monitoring for more accurate metabolic profiling.

This includes investigating why older adults might struggle to maintain adequate nutrition and developing targeted solutions that align with their needs and capabilities. The session emphasized the importance of behavioral change support, recognizing that successful interventions must be scientifically sound and practically implementable. This includes finding effective ways to support people in improving their health and wellbeing through sustainable behavioral changes. Research outcomes in nutrition and brain health interventions show significant heterogeneity, complicating comparing results across different studies and interventions. To address this challenge, the concept of Core Outcome Sets (COS) [151] has been proposed as a standardized minimum set of outcomes that should be measured and reported in clinical trials. While COS already exists for older adults with malnutrition, questions remain about its validity for outcome measurements. This standardization approach could help improve the comparability of research results and enhance the overall productivity of research groups by providing a consistent framework for outcome assessment and reporting.

Patients and caregivers may have varying beliefs and expectations about the effectiveness of the interventions, which can impact their participation and adherence. Maintaining a new diet or adhering to a supplementation regimen can be challenging for some participants, especially with the need to make long-term lifestyle adjustments.

## Conclusions and recommendations

The workshop recommendations are summarized in Table 1. Evidence presented demonstrates that nutrition plays a pivotal role in brain health and dementia prevention. The Mediterranean and MIND diets have shown particularly promising results, with the latter associated with up to a 53% reduction in AD risk when strictly followed [103]. Future research should prioritize several key areas. There should be emphasis on methodology, as there is a pressing need for standardized cognitive assessment methods and outcome measures to enable better comparison across studies. Developing COS specifically for nutrition interventions in brain health research would significantly advance the field. Future dietary intervention studies should focus investigation of the gut-brain axis requires, particularly regarding the role of microbiota in cognitive health and the potential of targeted interventions through pre-and probiotics. The field must move toward more personalized nutrition strategies that explicitly account for individual factors such as *APOE* genotype, baseline nutritional status, gut microbiome composition, and metabolic profile, in order to tailor interventions for maximum effectiveness and enhanced cognitive resilience [152–154]. There is a research need to study the mechanisms behind individual variation in response to nutritional interventions and investigate the optimal timing and duration of interventions across different age groups. We need to design long-term studies examining preventive interventions in younger populations particularly midlife. Long-term studies examining the impact of early-life nutrition on later cognitive outcomes are essential, building on the Barker’s hypothesis framework [155]. The success of multi-domain interventions like the FINGER trial [105] suggests that future research should integrate dietary modifications with other lifestyle factors. Priority should be given to developing practical, sustainable interventions that work at a population level while remaining adaptable to individual needs and cultural contexts. Research needed to determine effective low agency approaches to help populations shift toward plant-based diets and what impact this has on brain health and dementia risk (which can also positively impact on planetary health).

**Table 1:** Summary of evidence and future research recommendations to explore the nexus between nutritional and dietary factors with brain health for dementia prevention

	Summary of Evidence	Recommendations
Patient and Public Involvement and Engagement (PPIE)	Research should apply co-production methods to develop and test tools, interventions, where possible, with support from patients, carers and healthcare professionals. Co-producing a research project is an approach in which researchers, practitioners and the public work together, sharing power and responsibility from the start to the end of the project, including the generation of knowledge. **Key message**: Make co-production the default process for decision-making that impacts care to embrace research needs to include adapting research processes for accessibility, involving diverse voices, and formally evaluating the impact of PPIE.	Develop validated tools or bio-markers of nutrient intake that do not rely on recall methodology. Develop co-production toolkit to support researchers to engage with people with dementia and their carers/healthcare providers. Ensure equality and diversity in representing patients, carers and healthcare professionals. **Action**: UK Research and Innovation, civil society organisations giving a voice to marginalised groups; researchers, healthcare providers and citizens.
Biological Mechanisms	Nutritional factors impact brain health through multiple mechanisms including inflammation, oxidative stress, and gut microbiota modulation. Peripheral amyloid metabolism significantly influences Alzheimer’s disease progression, with impaired clearance of amyloid-beta potentially causing cerebral deposition. Postprandial lipid metabolism, especially in *APOE4* carriers, affects amyloid trafficking and blood-brain barrier integrity, with high saturated fat consumption potentially compromising clearance pathways. Mediterranean-style diets may support lipid homeostasis and amyloid clearance. However, evidence primarily comes from preclinical and observational studies, with personalized nutrition approaches based on *APOE* genotype requiring further investigation. **Key message**: Understanding the role of nutrition-based mechanism and approaches linked to *APOE* genotype.	Develop harmonized methodologies for dietary intake assessment, postprandial metabolic analysis, and neurovascular biomarkers. Prioritise large-scale longitudinal and interventional trials assessing how dietary components influence amyloid metabolism, neurovascular function, and cognitive decline in *APOE*-stratified cohorts. Utilise controlled dietary interventions, stable isotope-labelled lipid and amyloid studies, and Mendelian randomization approaches to establish direct links between food intake, peripheral amyloid metabolism, and Alzheimer’s risk. Investigate how gut-derived metabolites, particularly short-chain fatty acids and bile acids, influence peripheral and central Aβ metabolism, BBB integrity, and neuroinflammation. **Action**: Research evidence generators, research funders.
Population-Based Research	There is a lack of co-designed dietary interventions that address the accessibility and availability of nutritious and affordable food choices in remote and rural populations. For example, food hubs offer social, environmental and economic benefits to local communities. Population studies strongly link balanced diets to improved overall health and reduced chronic disease risk, including cardiovascular and cerebrovascular conditions. However, evidence connecting dietary patterns specifically to cognitive outcomes remains less robust. This research gap stems from methodological limitations in longitudinal studies, including absence of baseline cognitive assessments and infrequent nutritional data collection during follow-up periods. Additionally, participants often modify their dietary patterns after receiving diagnoses of risk factors, complicating long-term analysis. Changes in diet over time present challenges for extended studies tracking cognitive outcomes. Despite these limitations, existing epidemiological evidence suggests healthy dietary patterns may positively influence cognitive function, though more targeted research is needed. **Key message**: Focusing on brain health across the lifespan and embracing technology advances (e.g. genomics, imaging, and other technologies) to enhance data collection and analysis in population-based studies, leading to a deeper understanding of the biological mechanisms underlying brain health.	Future studies on population level evidence may benefit from utilising up and coming research methods for example– artificial intelligence and data linkage methods linking health records, dietary pattern (purchasing pattern) and lifestyle and social circumstances. The link between long term impact of diet and nutrition on brain structure and function and the gut-brain axis may be further explored using advanced neuro-imaging techniques and population cohorts may serve as valuable sampling framework as they captured various lifestyle, socioeconomic and dietary factors over years. Co-design place-based solutions to improve the food environment in remote and rural communities. Develop strategies to scale up food hubs and support a transition away from emergency food provision. Research needed to determine effective low agency approaches to help populations shift toward plant-based diets and what impact this has on brain health and dementia risk. **Action**: National government, research funders, research evidence generators with patients/carers.
Nutrient-Based Interventions	Current evidence supports key nutrients in dementia prevention including omega-3 fatty acids (DHA), B vitamins (B12, B6, folate), antioxidants (C, E), flavonoids, polyphenols, and vitamin D. Research indicates combined nutrient approaches are more effective than single-nutrient interventions for cognitive protection. Methodological challenges include poor trial design, inadequate control of confounding factors, and limitations in dietary assessment methods. The *APOE* genotype emerges as crucial in determining intervention responses, with supplementation showing greatest benefit in those with poorer nutritional status. **Key message**: Focus on understanding the effectiveness of specific nutrients and dietary patterns for preventing or delaying cognitive decline and dementia. Additionally, research needs to explore the efficacy of interventions like the Mediterranean diet and the MIND diet, which incorporate neuroprotective foods. More robust clinical trials are needed to assess the effects of these interventions on cognitive outcomes and to address inconsistencies in current research.	Develop standardised protocols for nutrient intervention trials, incorporating precision nutrition approaches and genetic profiling. Create improved dietary assessment tools specific to cognitive health research, focusing on prospective rather than retrospective methodologies. Investigate synergistic effects between multiple nutrients and dietary patterns. Design long-term studies examining preventive interventions in younger populations particularly midlife. Establish validated biomarkers for early cognitive decline and intervention effectiveness for *APOE* genotype. Research the interaction between *APOE* genotype and specific nutrients. Study the mechanisms behind individual variation in response to nutritional interventions. Investigate the optimal timing and duration of interventions across different age groups. Research the impact of dietary patterns on specific cognitive domains rather than general cognition. **Action**: National government, research funders, research evidence generators with patients/carers.
Dietary Patterns and Multi-Modal Interventions	Dietary patterns generally outperform single-component approaches for cognitive health. Mediterranean-style dietary interventions show mixed cognitive outcomes across multiple trials, with some demonstrating improvements while others report less convincing findings. Evidence suggests caloric restriction itself may benefit cognition regardless of specific dietary pattern. Multi-domain interventions combining diet modifications with exercise, cognitive training, and vascular risk management have established the strongest evidence base for preventing cognitive decline. Global adaptations of these comprehensive approaches demonstrate promising results across diverse populations and cultural contexts. Studies combining dietary modifications with exercise interventions highlight intervention sustainability and improved adherence over time, with potential additional benefits. **Key message**: Focus on understanding which dietary factors, including specific nutrients and foods, are most effective in preventing or delaying dementia. Further research is also needed to determine whether nutritional needs differ between the disease stages, and whether dietary interventions can be tailored to individual needs and preferences.	Define optimal dietary patterns for brain health across different populations. Adapt the FINGER model for diverse countries to create feasible preventive strategies for various at-risk groups. Study effective dosing and delivery methods for multimodal interventions. Develop recruitment strategies to evaluate dietary interventions in ethnic/racial minorities and lower socioeconomic communities. Identify best approaches for supporting long-term behaviour change. Use AI and digital health technologies to promote dietary changes and improve cognitive performance. Assess gut-microbiome interventions in healthy participants experiencing cognitive challenges or chronic conditions to identify intervention windows and estimate effect magnitudes. Select cognitive domains based on specific cognitive factors and study populations. **Action**: UK Research and Innovation, National government, research funders, research evidence generators with patients/carers.:

Special attention should be paid to vulnerable populations, including those in rural areas, undernourished adults and women, particularly regarding pregnancy-related impacts on brain health. This includes developing recruitment strategies to evaluate dietary interventions in ethnic/racial minorities and lower socioeconomic communities. Incorporating lived experience in study design and implementation will be crucial for ensuring effective, practical interventions that are culturally adapted. For example, there is a research gap to co-design place-based solutions to improve the food environment in remote and rural communities. The development of apps and AI may facilitate more remote and rural research, where novel tools and bio-markers can assist with data collection. There is a need for improved dietary assessment tools specific to cognitive health research, focusing on prospective rather than retrospective methodologies. A toolkit to encourage and support the capture the lived experience of patients, caregivers and healthcare providers would be helpful.

Finally, research should explore innovative assessment methods, including the use of artificial intelligence for identifying effective interventions and biomarker discovery. Developing early detection methods and preventive strategies, particularly through the gut-brain axis, represents a promising direction for future investigation.

## Electronic supplementary material

Below is the link to the electronic supplementary material.

Supplementary Material 1

## Data Availability

No datasets were generated or analysed during the current study.
