# Supplementary material for: Consensus statement on exploring the Nexus between nutrition, brain health and dementia prevention
Source: Nutr Metab (Lond). 2025 Jul 25;22:82. doi: 10.1186/s12986-025-00981-6 (PMC12291389; doi:10.1186/s12986-025-00981-6)
Supplement: Supplementary file 1 — Supplementary Material 1 [file 12986_2025_981_MOESM1_ESM.docx]

**Consensus Statement on Exploring the Nexus Between Nutrition, Brain Health and Dementia Prevention. Johnstone *et al.***

**Appendix 1 of the Supplementary Material**

Workshop Agenda

Exploring the Nexus Between Nutrition, Brain Health, and Dementia Prevention

Venue: The Rowett Institute, University of Aberdeen, Foresterhill Health Campus, Ashgrove Road West, Aberdeen

Day 1, 27^th^ June 2024

 9:00-9:30am Coffee, Registration and informal introductions

 9:30-9.45am Welcome from School Research Director, Prof. Ian Stansfield

9:45-10.00am Overview of the Workshop Objectives and Agenda – Prof Alex Johnstone and Prof Mario Siervo

10:00-10:45 Session 1: The Lived Experience of Ageing and Dementia (PPI Group)

Speakers: MA and ER

Coffee & comfort break (10.45-11am)

11:00 –12.00 Session 2: Mechanisms Linking Nutrition to Brain Health

 2 x 15min talks plus workshop of 30 mins (Discussion Focus: Methods and technologies for nutrition and brain research)

Speakers: Prof John Mamo and Dr David Vauzour

12.00-13.00 Session 3: Nutritional Epidemiology and Brain Health

2 x 15min talk plus workshop of 30 mins (Discussion Focus: Nutritional Epidemiology for Dementia Prevention: What’s next? )

Speakers: Prof Emiliano Albanese and Dr Catherine Hughes

Buffet Lunch (1-2pm)

14:00-15.00 Session 4: Food-Gut-Brain Axis for Healthy Ageing

2 x 15min talks plus workshop of 30 mins (Discussion Focus: Precision medicine and personalised nutrition for dementia prevention)

Speakers: Prof Alex Johnstone and Dr Stephanie Grabrucker

Coffee & comfort break (15.00-15:30pm)

15.30-16.30 Session 5: Identifying Future Research Priorities Using CO-CREATE Tool

Leaders: Prof Alex Johnstone and Dr Dan Crabtree (2 groups)

16.30 – 17.00 Session 6: Case Study: Active Research in Aberdeen

Speakers: Prof Phyo Myint and Prof Jenna Gregory

7.30pm Dinner

Day 2, 28^th^ June 2024

8.00am Optional Tour of Rowett Institute

8.45 – 9.00am Coffee & Welcome, Dean for Impact and Engagement, Prof. Michelle MacLeod

9.00 – 9.45 Session 7: ECR session – elevator pitches

Speakers: Dr Daniel Crabtree, Dr Adrian Holliday, Dr Boushra Dalile and Dr Leticia Radin Pereira

9.45-10:45 Session 8: Nutrient-Based Interventions and brain health

2 x 15min talks plus workshop of 30 mins (Discussion Focus: Nutrient-Based intervention studies for dementia prevention)

Speakers: Prof Mario Siervo and Prof Giuseppe Grosso

Coffee & comfort break 10:45-11:00am

11.00-12.00 Session 9: Dietary Patterns for Brain Health

2 x 15min talks plus workshop of 30 mins (Discussion Focus: Trans-disciplinary research for multi-dimensional intervention strategies)

Speakers: Prof. Catherine Itsiopoulos and Dr Claire McEvoy

 12:00-12:30pm Session 10: Update from funders  Ms Anne McGavigan, School of Medicine, Medicaal Sciences and Nutrition

12.30 – 12.45 Summary and Final Remarks and Future Plans

Mario & Alex

Lunch (12:45-1:30pm)

End of Meeting, depart.
